# 3D printed templates improve the accuracy and safety of pedicle screw placement in the treatment of pediatric congenital scoliosis

**DOI:** 10.1186/s12891-021-04892-4

**Published:** 2021-12-04

**Authors:** Jun Cao, Xuejun Zhang, Haonan Liu, Ziming Yao, Yunsong Bai, Dong Guo, Lei Feng

**Affiliations:** Beijing Children’s Hospital, Capital Medical University, National Center for Children’s Health, #56 Nan Li Shi Road, Xi Cheng District, Beijing, 100045 China

**Keywords:** Pediatric congenital scoliosis, 3D printed template, Computer-assisted surgery, Screw placement

## Abstract

**Background:**

Three-dimensional (3-D) printed guidance templates are being increasingly used in spine surgery. The purpose of this study was to determine if 3D printed navigation templates can improve the accuracy of pedicle screw placement and decrease the complication rate compared to freehand screw placement in the treatment of children with congenital scoliosis.

**Methods:**

The records of pediatric patients with congenital scoliosis treated at our hospital from January 2017 to January 2019 were retrospectively reviewed. Patients were divided into those where a 3D printed guidance templated was used and those in which the freehand method was used for pedicle screw placement. The accuracy rate of pedicle screw placement, surgical outcomes, and complications were compared between groups.

**Results:**

A total of 67 children with congenital scoliosis were included (43 males and 24 females; mean age of 4.13 ± 2.66 years; range, 2–15 years). There were 34 children in the template-assisted group and 33 in the freehand group. The excellent accuracy rate of pedicle screw placement was significantly higher in the template-assisted group (96.10% vs. 88.64%, *P* = 0.007). The main Cobb angle and kyphosis angle were similar between the 2 groups preoperatively and postoperatively (all, *P* > 0.05), and in both groups both angles were significantly decreased after surgery as compared to the preoperative values (all, *P* < 0.001). The degree of change of the Cobb angle of the main curve and kyphosis angle were not significantly different between the 2 groups. There were no postoperative complications in the template group and 4 in the freehand group (0% vs. 12.12%; *P* = 0.009). All 4 patients with complications required revision surgery.

**Supplementary Information:**

The online version contains supplementary material available at 10.1186/s12891-021-04892-4.

## Introduction

Congenital scoliosis is defined as a lateral spinal curvature of > 10%, and accounts for approximately 10% of pediatric spine deformities [1]. The prevalence of congenital scoliosis is estimated to be approximately 0.5 to 1 per 1000 live births [2]. The etiology of congenital scoliosis is not fully understood, but failure of formation and or segmentation during somitogenesis is believed to be part of the pathogenic mechanism [3]. Congenital scoliosis has a serious impact on spinal growth [4], and up to 85% of patients with congenital scoliosis develop a final curve >41^o^ after 10 years of age if not treated [5–7].

Surgery is the primary treatment for most cases of congenital scoliosis, and pedicle screw instrumentation has been widely adopted for the treatment of scoliosis because it is associated with superior correction efficacy [8, 9]. Currently, the freehand method of screw placement with fluoroscopic guidance is the most commonly used method of screw placement [10]; however, the method is associated with a considerable risk of pedicle screw misplacement [11, 12].

With the development of three-dimensional (3-D) printing technology, 3D printed guide templates have become commonly used in spine surgeries such as those for post-traumatic injury, degenerative or inflammatory diseases, pathology of the cranio-cervical junction, and idiopathic scoliosis [13]. Tan et al. reported that the use of a 3D printed spine model improved the safety of pedicle screw placement in the treatment of severe spine deformities [14]. A recent systematic review and meta-analysis both concluded that 3D printed guidance templates improved the accuracy of pedicle screw placement [15, 16]. However, these studies primarily included adult patients; few studies have examined the use of 3D printing techniques in the treatment of pediatric congenital scoliosis [10].

Thus, the purpose of this study was to determine if the use of 3D printed navigation templates can improve the accuracy of pedicle screw placement and decrease the complication rate compared to the freehand screw placement method and still achieve an adequate treatment effect in the treatment of children with congenital scoliosis.

## Methods

### Study design and subjects

In this study, the records of pediatric patients with congenital scoliosis treated at our hospital from January 2017 to January 2019 were retrospectively reviewed. Inclusion criteria were: 1) Congenital scoliosis; 2) Comprehensive evaluation resulting in a recommendation for surgical treatment; 3) No prior history of spine surgery; 4) A minimum follow-up period of 12 months; and 5) < 10 years of age. Patients with non-congenital scoliosis were excluded from the analysis. Idiopathic, neuromuscular, syndrome.

Patients were divided into a template-assisted group and a freehand screw placement group. In the template assisted group, a 3D printed anatomical model of the deformed spine was made using preoperative computed tomography (CT) scan data. The model was then used surgical planning and optimization of screw placement. In the freehand group, the conventional freehand technique was used for intraoperative screw placement.

This study was approved by the institutional review board (IRB) of our hospital. The requirement of written informed consent was waived by the IRB due to the retrospective nature of this study.

### Design and printing of 3D navigation templates

Patient CT data was converted to the Digital Imaging and Communications in Medicine (DICOM) format file using the Picture Archiving and Communication Systems (PACS) of our hospital. The data were then imported into Mimics version 19.0 software (Materialise, USA) (Supplementary Fig. 1). The reconstructed model of the spine was produced using 3D reconstruction calculations. Four, 2.2-mm diameter cylinders were generated using the Analyze module to simulate Kirschner wires for screw placement. The ideal path of the posterior pedicle screw was preliminarily designed (Supplementary Fig. 2A), and the cylinders and the reconstructed model were fitted together (Supplementary Fig. 2B).

The model (STL format) was imported into 3-Matics software (Materialise, USA). Positioning sites for the guide plates were selected on the surface of the posterior spinous process, lamina, and lateral mass of the vertebral body, and the guide plates were then generated (Supplementary Fig. 3A).

After satisfactory generation of all local guide plates, they were integrated into a complete navigation template (Supplementary Fig. 3B). Navigation template data (STL format) were imported into 3D printing pre-processing software (Magics 20.03; Materialise, USA) for corrections (Supplementary Fig. 4). The data were then imported IdeaMaker version 3.1.3 software (Raise3D, Inc., Shanghai, China) (line width = 0.4 mm, layer thickness = 0.25 mm, support angle = 54 ^o^). The resulting gcode file was then printed using a Pro2 plus 3D printer (Raise3D, Inc.). The model was printed using polylactic acid (PLA) with a nozzle temperature of 215 °C and a hot bed temperature of 60 °C. After printing, a 2.0-mm Kirschner wire was used to clear the pinholes reserved on the printed navigation template.

### Surgical procedure

All surgeries were performed using a posterior surgical approach. After induction of anesthesia and endotracheal intubation, the patient was placed in the prone position and an incision was made along the middle of the posterior spine. The paravertebral muscles were separated to expose the spinous processes, lamina, upper and lower articular processes, and transverse processes.

In the template-assisted group, the sterilized navigation template was firmly attached to the spine via the lamina. An electric drill was used to drill pinholes for 0.5-mm diameter Kirschner wires using the pinholes on the navigation template. After correct position of the Kirschner wires was confirmed by fluoroscopy the pedicle screws were placed. In the freehand group, placement of pedicle screws was estimated based on the anatomical features of the exposed lamina and articular processes. Kirschner wires were then placed in the corresponding positions, and after correct positioning of the Kirschner wire was confirmed by fluoroscopy the pedicle screw was placed.

In both groups, after pedicle screw placement the semi-vertebral body was excised and orthopedically fixed, followed by placement of autogenous bone graft. Once completed, the incision was closed in layers. Postoperative CT was performed to determine the accuracy of pedicle screw placement and degree of correction.

### Outcomes

Data extracted from the medical records included age and sex, the degree of preoperative deformity, operation time, intraoperative blood loss, and postoperative complications. The postoperative CT data were used to evaluate the accuracy of pedicle screw placement based on the Kawaguchi method [17]. Pedicle screw placement was designated as Grade 0 to 3. Grade 0: The pedicle screw was completely inside the pedicle; Grade 1: The pedicle screw partially penetrated the medial or lateral pedicle < 2 mm; Grade 2: The pedicle screw partially penetrated the medial or lateral pedicle between 2 and 4 mm; Grade 3: The pedicle screw partially penetrated the medial or lateral pedicle > 4 mm. The excellent pedicle screw placement accuracy rate and the good accuracy rate were defined as follows:

$$\mathrm{Excellent}\ \mathrm{accuracy}\ \mathrm{rate}\ \left(\mathrm{Grade}\ 0\right)=\left[\mathrm{Number}\ \mathrm{of}\ \mathrm{Grade}\ 0\ \mathrm{screws}\ \mathrm{placed}/\mathrm{the}\ \mathrm{total}\ \mathrm{number}\ \mathrm{of}\ \mathrm{screws}\ \mathrm{placed}\right]\times 100$$

$$\mathrm{Good}\ \mathrm{accuracy}\ \mathrm{rate}\ \left(\mathrm{Grade}\ 0+\mathrm{Grade}\ 1\right)=\left[\mathrm{Number}\ \mathrm{of}\ \mathrm{Grade}\ 0+\mathrm{Grade}\ 1\ \mathrm{screws}\ \mathrm{placed}/\mathrm{the}\ \mathrm{total}\ \mathrm{number}\ \mathrm{of}\ \mathrm{screws}\ \mathrm{placed}\right]\times 100$$

### Statistical analysis

Continuous data were presented as mean ± standard deviation (SD). Independent comparisons between 2 groups were made using Student’s independent t-test, and the paired t-test was used for repeated measurements. If normality of continuous data was not assumed, the Mann-Whitney U test and Wilcoxon signed-rank test were used for comparisons. Categorical variables were presented as number and percentage, and were compared using the chi-square test or Fisher’s exact test if an expected value ≤5 was found. Two-way mixed-design ANOVA was used to investigate the differences of Cobb angle of the main curve and angle of kyphosis before and after surgery. Linear regression analysis was used to investigate the associations of factors related to change of Cobb angle and kyphosis. All statistical analyses were performed using IBM SPSS version 25 software (SPSS Inc., IBM Corporation, Somers, New York). All analyses were 2-tailed, and a value of *P* < 0.05 was considered to indicate statistical significance.

## Results

### Patients

A total of 67 children with congenital scoliosis were included in the analysis. There were 43 males and 24 females with a mean age of 4.13 ± 2.66 years (range, 2–15 years). There were 34 children in the template-assisted group and 33 in the freehand group. Patient demographic and clinical characteristics are summarized in Table 1. There was no significant difference in age, sex, scoliosis types, combined malformation, malformation type, scoliosis location, and scoliosis side between the 2 groups (all, *P* > 0.05). All operations were performed by the same senior orthopedic surgeon and the first author.

**Table 1:** Patient demographic and clinical characteristics

	Template-assisted group (*n* = 34)	Freehand group (*n* = 33)	All (*N* = 67)	*P*
Age, years	4.06 ± 2.28	4.21 ± 3.04	4.13 ± 2.66	0.816
Sex				0.548
Male	23 (67.65)	20 (60.61)	43 (64.18)	
Female	11 (32.35)	13 (39.39)	24 (35.82)	
Scoliosis type				0.206
Defect of formation	12 (35.29)	18 (54.55)	34 (50.75)	
Defect of segmentation	2 (5.88)	2 (6.06)	4 (5.97)	
Mixed	20 (58.82)	13 (39.39)	33 (49.25)	
Combined malformation				0.323
No	22 (64.71)	25 (75.76)	47 (70.15)	
Yes	12 (35.29)	8 (24.24)	20 (29.85)	
Malformation type				0.067
Single	13 (38.24)	20 (60.61%)	33 (49.25)	
Multiple	21 (61.76	13 (39.39)	34 (50.75)	
Scoliosis side				0.216
Left	18 (52.94)	18 (54.55)	36 (53.73)	
Right	16 (47.06)	13 (39.39)	29 (43.28)	
Kyphosis	0	2 (6.06)	2 (2.99)	

Data are presented as mean ± standard deviation, or count (percentage)

### Surgical outcomes

Surgical data and outcome measures are summarized in Table 2. Surgery was successful in all cases, and the operation time and amount of intraoperative bleeding were similar between the 2 groups (both, *P* > 0.05). However, there were a significantly greater number of screws placed and levels fused in the template-assisted group than the freehand group (both, *P* < 0.05).

**Table 2:** Surgical data

	Template-assisted group (*n* = 34)	Freehand group (*n* = 33)	All (*N* = 67)	*P*
Surgical time, min	160.44 ± 46.03	156.82 ± 55.21	158.66 ± 50.40	0.771
Intraoperative bleeding, ml	368.53 ± 254.64	306.15 ± 232.24	337.81 ± 244.05	0.299
Screw placement outcomes				
Number of screws	6.59 ± 2.72	5.24 ± 2.05	5.93 ± 2.49	0.026
Excellent accuracy rate %	96.10 ± 7.02	88.64 ± 13.91	92.42 ± 11.51	0.007
Good accuracy rate, %	99.02 ± 3.28	97.14 ± 7.20	98.09% ± 5.60	0.171
Number of fused levels	3.47 ± 1.58	2.70 ± 1.13	3.09 ± 1.42	0.025
Cobb angle main curve, °				
Preoperative	38.77 ± 13.59	32.91 ± 11.87	35.88 ± 13.02	0.065
Postoperative	8.94 ± 9.20^a^	5.86 ± 7.30^a^	7.42 ± 8.40	0.135
Kyphosis angle, °				
Preoperative	20.33 ± 14.77	19.68 ± 16.17	20.01 ± 15.36	0.864
Postoperative	8.87 ± 7.45^a^	4.70 ± 9.56^a^	6.82 ± 8.75	0.073
Complications	0	4 (12.12)	4 (6.00)	0.009

Data are presented as mean ± standard deviation, or count (percentage)

^a^Both the Cobb angle of the main curve and kyphosis angle were significantly decreased after surgery in both groups (both, *P* < 0.001)

Excellent accuracy rate (Grade 0) = [Number of Grade 0 screws placed / the total number of screws placed] × 100

Good accuracy rate (Grade 0 + Grade 1) = [Number of Grade 0 + Grade 1 screws placed / the total number of screws placed] × 100

The excellent accuracy rate of pedicle screw placement was significantly higher in the template-assisted group than in the freehand group (96.10% vs. 88.64%, *P* = 0.007). The good accuracy rate of pedicle screw placement was similar between the 2 groups (99.02% vs. 97.14%, *P* = 0.171) (Table 2). The distributions of pedicle screw perforation grades in the 2 groups is shown in Table 3.

**Table 3:** Distributions of pedicle screw perforation grades in the 2 groups

Perforation grade	Template-assisted group	Freehand group
Grade 0	212	152
Grade 1	13	15
Grade 2	3	4
Grade 3	0	2

Data are presented as count

Pedicle screw perforation grading. Grade 0: The screw is located within the pedicle. Grade 1: Perforation < 25% of the screw diameter is present. Grade 2: Perforation of 25 to 50% of the screw diameter. Grade 3: Perforation > 50% of the screw diameter

### Clinical outcomes

The main Cobb angle and kyphosis angle were similar between the 2 group preoperatively and postoperatively (all, *P* > 0.05, Table 2). However, in both groups the main Cobb angle and kyphosis angle were significantly decreased after surgery as compared to the preoperative values (all, *P* < 0.001). The degree of change of the Cobb angle of the main curve (− 29.84 ± 10.88° vs. -27.05 ± 10.77°; *P* = 0.295) and kyphosis angle (− 11.46 ± 15.11° vs. -14.98 ± 16.20°; *P* = 0.361) were not significantly different between the 2 groups.

Linear regression analysis of factors related to the change of Cobb angle of the main curve and kyphosis angle showed no significant differences between the groups (both, *P* > 0.05), indicating a comparative treatment effect between the 2 groups.

**Fig. 1:**
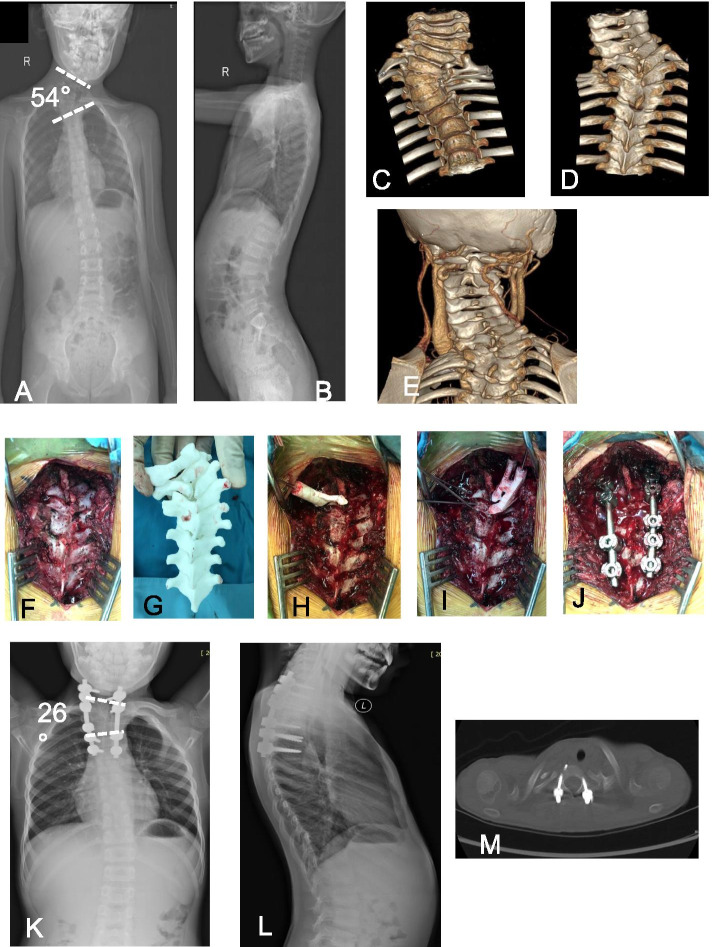
A 7-year-old male was seen due to worsening neck skew. **A, B** X-rays showed congenital scoliosis and multiple vertebral deformities in the cervical and upper thoracic spine. The angle of scoliosis was about 72°, and the trunk was offset by 2.7 cm. **C-E** Computed tomography (CT) showed multiple vertebral malformations in the upper thoracic spine. CT angiography (CTA) was performed to determine the position of the carotid artery. **F-J** The spine was exposed during the operation, and a premade 3D printed navigation template was placed on the spine. The spinous processes and transverse processes were used as anatomical landmarks to confirm the fixation position. Using the navigation template Kirschner wires were placed, followed by placement of pedicle screws. **K-L** Postoperative anterolateral radiographs of the spine showed correction of scoliosis to 26°. M) Postoperative CT showed that all of the screws were placed in the center of the pedicles

**Fig. 2:**
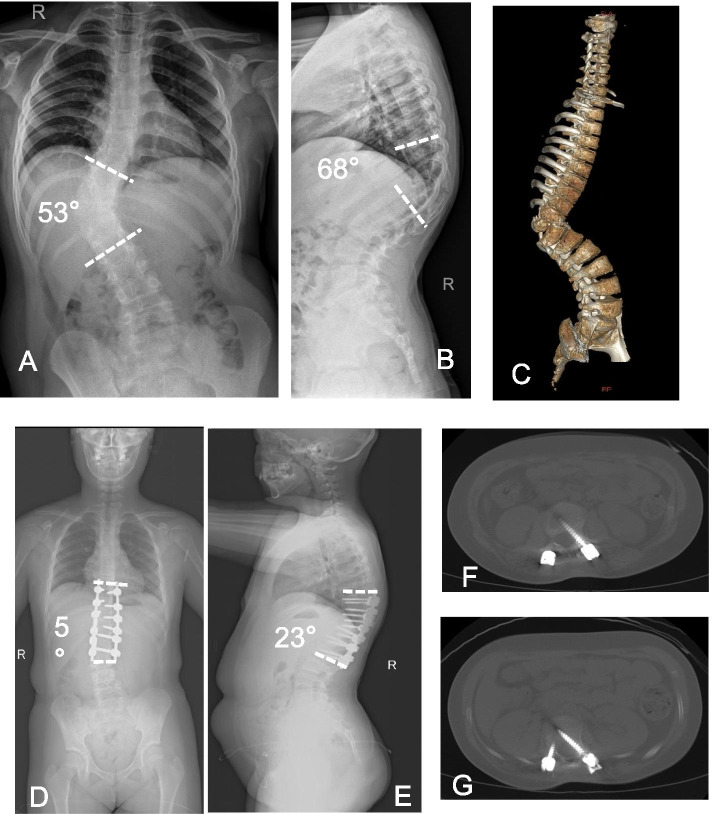
A 9-year-old female patient with a bulging back. **A-C** X-ray showed congenital scoliosis and a thoracolumbar hemi-vertebral deformity. The scoliosis angle was about 53° and kyphosis angle was 68°. **D, E** Postoperatively, the scoliosis angle was corrected to 5° and the kyphosis angle to 23°. After the operation, the patient’s lower limb muscle strength decreased but returned to normal after 1 month. **F, G** Computed tomography (CT) showed that the left L1 and L2 pedicle screws were offset into the spinal canal

Images of representative cases of the template-assisted group are shown in Figs. 1 and 2.

### Postoperative complications

The postoperative complication rate was significantly lower in the template-assisted group than in the freehand group (0% vs. 12.12%; *P* = 0.009). No patients in the template-assisted group experienced postoperative complications compared to 4 patients in the freehand group. In the freehand group, 2 patients had transiently reduced muscle strength after surgery, and 2 patients had internal fixation-related complications due to the pedicle screw not being in the middle of the pedicle and thus penetrating the outer wall of the pedicle resulting in splitting of the pedicle. The patients with internal fixation-related complications required revision surgery and ultimately had good outcomes. Muscle strength in the other 2 patients gradually returned after approximately 1–2 months.

## Discussion

The results of this study showed that the use of a 3D printed template of the spine can improve the accuracy or pedicle screw placement and reduce complications when used for the treatment of pediatric patients with congenital scoliosis as compared to the freehand method. Clinical outcomes with respect to correction of Cobb angle and kyphosis were not different than when the freehand method was used. Current screw placement methods include freehand, the use of a navigation system [18], and robot-assisted pedicle screw placement [19], and the conventional freehand method is associated with low accuracy of screw placement [11, 12].

For patients with congenital scoliosis treated at an early age (3–5 years), hemi-vertebrae excision and short segment fusion may be performed when there is a single or 2-level congenital vertebral defect [20]. Short segment fusion may reduce the number of segments that are fixed; however, this is often associated with instrumentation complications. Especially in pediatric scoliosis patients, pedicle screw misplacement is more likely to occur due to the presence of small pedicles and severely rotated vertebrae, leading to severe complication such as injury to the nerve roots, spinal cord, and major vessels as well as pedicle fracture [21–23]. An intraoperative pedicle fracture requires the need of an additional fused level, and an unidentified pedicle fracture may lead to loss of postoperative correction and neurological complications. Ledonio et al. [24]. conducted a systematic review that included 13,536 pedicle screws placed in 1353 pediatric patients with spinal deformities, and found that the overall accuracy of pedicle screw placement by the freehand technique was 94.5%; however, the accuracy decreased with more severe spinal deformities.

3D printing technology is increasingly being used for the production of guiding templates for screw placement in spinal operations [25, 26]. However, most studies have focused on adult patients; studies examining the use of 3D printed guiding templates in the treatment of congenital scoliosis are scarce. Recently, Vissarionov et al. [10] reported that the accuracy of screw placement using 3D printed guiding templates is more accurate than when placed using the freehand method for the treatment of congenital scoliosis (94.4% vs. 53.8%). Nevertheless, the study only compared the accuracy of screw placement; it did not examine the effectiveness of correction or postoperative complications.

In this study, we evaluated the accuracy and safety of 3D printed template-assisted screw placement in the treatment of congenital scoliosis. The results showed that the template-assisted group had significantly more screws placed and fused levels than the freehand group. The group that the 3D navigation templates were used with had more complex deformities and more severe scoliosis, which resulted in the use of more screws and a greater number of levels fused.

Our results showed no significant differences in the amount of intraoperative bleeding and surgical time between the 2 groups. This is mainly because corrective surgery for congenital scoliosis often requires hemivertebra resection, which requires a large amount of time and is associated with marked bleeding. We postulate that the reduction in surgical time and blood loss using the navigation templates were too small to affect the overall operation time and blood loss. On the other hand, a systematic review and meta-analysis by Yu et al. [16] that primarily included adult patients showed that use of a 3D printed drill guide template in the treatment of spine neurosurgery resulted in a shorter operation time and reduced intraoperative blood loss.

Although the good accuracy rate of pedicle screw placement was comparable between the 2 groups, the template-assisted group had a significantly higher excellent accuracy rate of pedicle screw placement as compared with the freehand group (96.10% vs. 88.64%, *P* = 0.007). As previously mentioned, Vissarionov et al. [10] also showed that using 3D printed navigation templates improved the accuracy of screw placement as compared to the freehand method (94.4% vs. 53.8%). Taken together, the results suggest that 3D printed navigation templates can significantly improve the accuracy of screw placement in the surgical treatment of congenital scoliosis. Congenital scoliosis is commonly treated with posterior lumbar fusion, and the large contact area between the posterior lamina and the navigation template assists in improving the accuracy of screw placement.

In this study, the Cobb angle of the main curve and kyphosis angle were significantly decreased after the operation in both groups; however, there were no significant differences between the 2 groups. This finding indicates that both methods are effective for the treatment of congenital scoliosis. However, there were no complications in the templated-assisted group, whereas there were 4 patients that experienced complications in the freehand group and all 4 patients required revision surgery.

As previously mentioned, current screw placement methods primarily include freehand placement, the use of an image-guided navigation system, and robot-assisted pedicle screw placement. Image-guided navigation systems and robotic screw placement result in much higher accuracy than the freehand method [27, 28]. However, both of these methods have high technical requirements including the equipment which is costly, surgeon technical expertise with the equipment, and with some systems an increase in the dosage of intraoperative radiation and operation time [18, 19, 27, 28]. For these reasons, we sought to develop a much simpler method—a 3D printed navigation template. While image-guided navigation and robotic screw placement result in the highest accuracy, we consider these methods in a different category because of the aforementioned points, and because of the technology involved other methods certainly will be associated with inferior accuracy. For this reason, we do not believe a direct comparison of the 3D printed navigation template image-guided navigation and robotic screw placement is a fair comparison; for these reasons we compared our method with the freehand method.

There are limitations of this study that require consideration. This was a retrospective study, and the number of patients was relatively small. The primary outcome was the accuracy of screw placement as assessed by postoperative CT. However, the follow-up duration was short with respect to the condition treated and much longer follow-up is necessary to determine the long-term rate of complications and treatment effect.

## Conclusions

In summary, our results showed that the use of a 3D printed navigation template to guide pedicle screw placement in the treatment of congenital scoliosis improves the excellent accuracy rate of screw placement and reduces the rate of postoperative complications as compared to the freehand method. Future studies are warranted to better determine the role and benefits of using 3D printed guidance templates in the treatment of congenital scoliosis.

## Supplementary Information

**Additional file 1: Supplementary Figure 1.** Computed tomography (CT) data were converted to DICOM files by the PACS system, and then were imported into Mimics 19.0 software. The reconstructed model was obtained through 3D reconstruction calculation. **Supplementary Figure 2.** Four 2.2-mm diameter cylinders were generated using the Analyze module to simulate Kirschner wires for screw placement. The ideal path of the posterior pedicle screws was preliminarily designed (A), and the cylinders and the reconstructed model were fitted together (B). **Supplementary Figure 3.** A) Data were imported into 3-Matics software, the sites for positioning sites for guide plates were selected on the surface of the posterior spinous processes, lamina, and lateral mass of the vertebral bodies, and the guide plates were generated. B) After all connections were made, all local guide plates were integrated into a navigation template. **Supplementary Figure 4.** Navigation template data in STL format were imported into Magics 20.03 3D printing pre-processing software for correction..

## Data Availability

All data generated or analyzed in this study are included in this published article.

## Abbreviations

3-D: Three-dimensional
CT: Computed tomography
DICOM: Digital Imaging and Communications in Medicine
PACS: Picture archiving and communication systems
SD: Standard deviation
