# Supplementary material for: 3D printed templates improve the accuracy and safety of pedicle screw placement in the treatment of pediatric congenital scoliosis
Source: BMC Musculoskelet Disord. 2021 Dec 4;22:1014. doi: 10.1186/s12891-021-04892-4 (PMC8645104; doi:10.1186/s12891-021-04892-4)

**Supplementary Figure 1.** Computed tomography (CT) data were converted to DICOM files by the PACS system, and then were imported into Mimics 19.0 software. The reconstructed model was obtained through 3D reconstruction calculation.


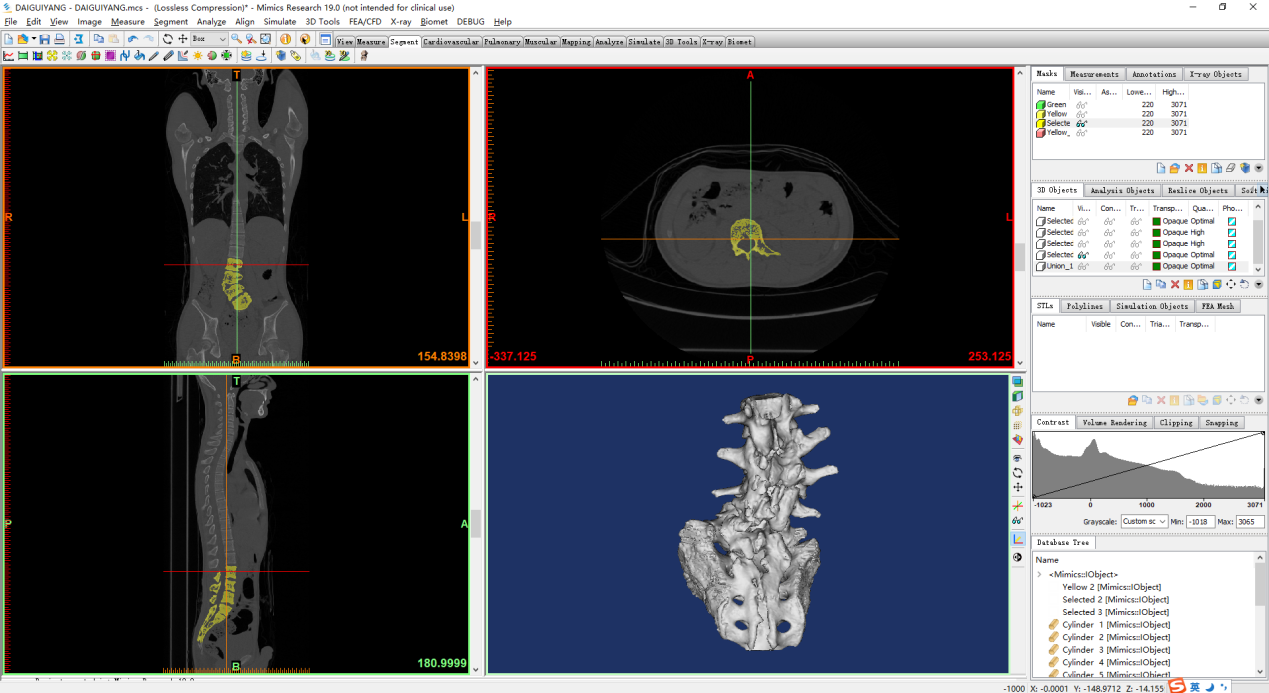


**Supplementary Figure 2.** Four 2.2-mm diameter cylinders were generated using the Analyze module to simulate Kirschner wires for screw placement. The ideal path of the posterior pedicle screws was preliminarily designed (A), and the cylinders and the reconstructed model were fitted together (B).

**A**


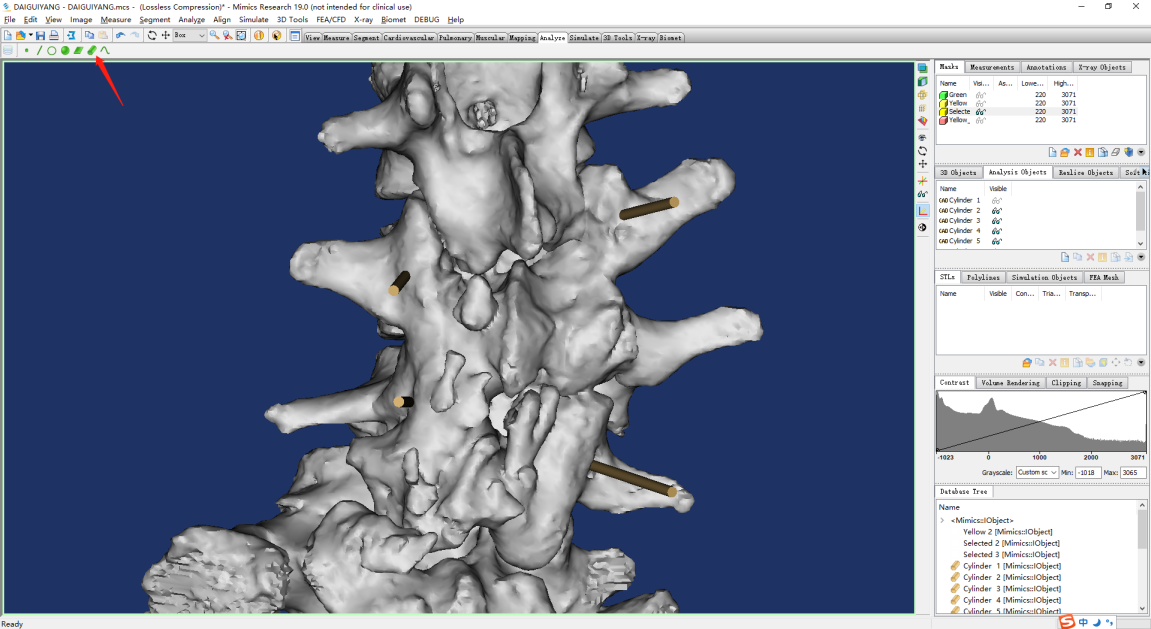


**B**


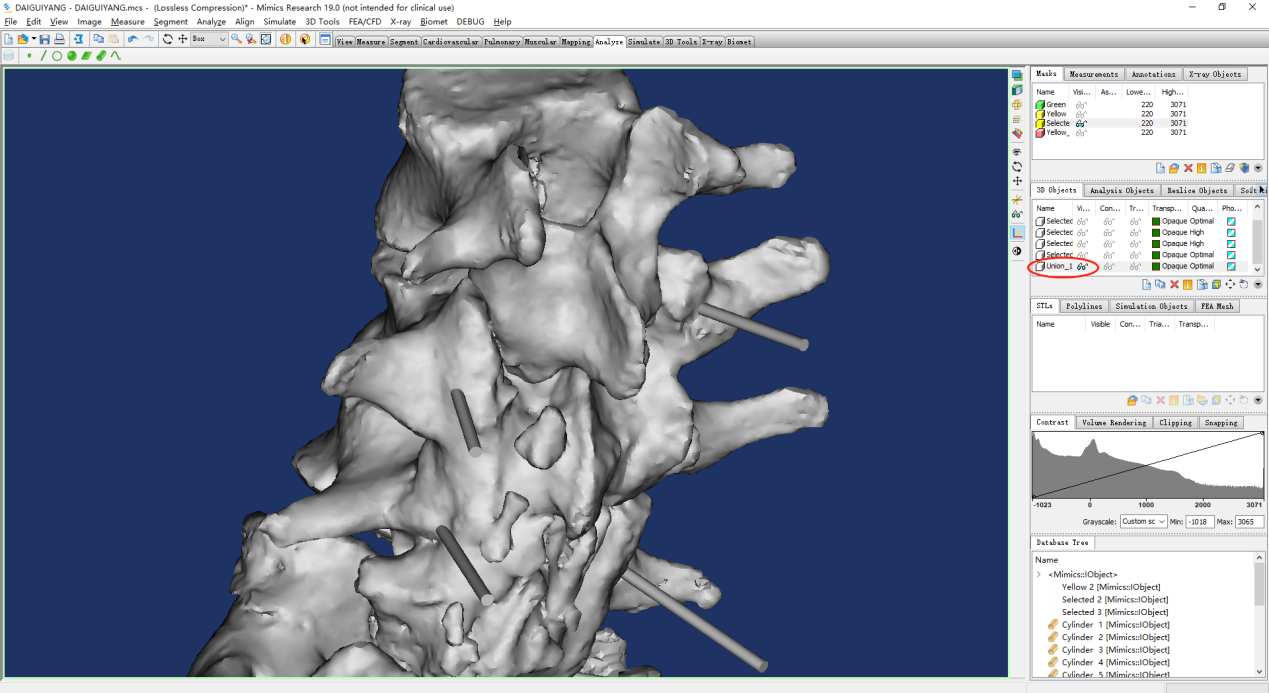


**Supplementary Figure 3.** A) Data were imported into 3-Matics software, the sites for positioning sites for guide plates were selected on the surface of the posterior spinous processes, lamina, and lateral mass of the vertebral bodies, and the guide plates were generated. B) After all connections were made, all local guide plates were integrated into a navigation template.

**A**


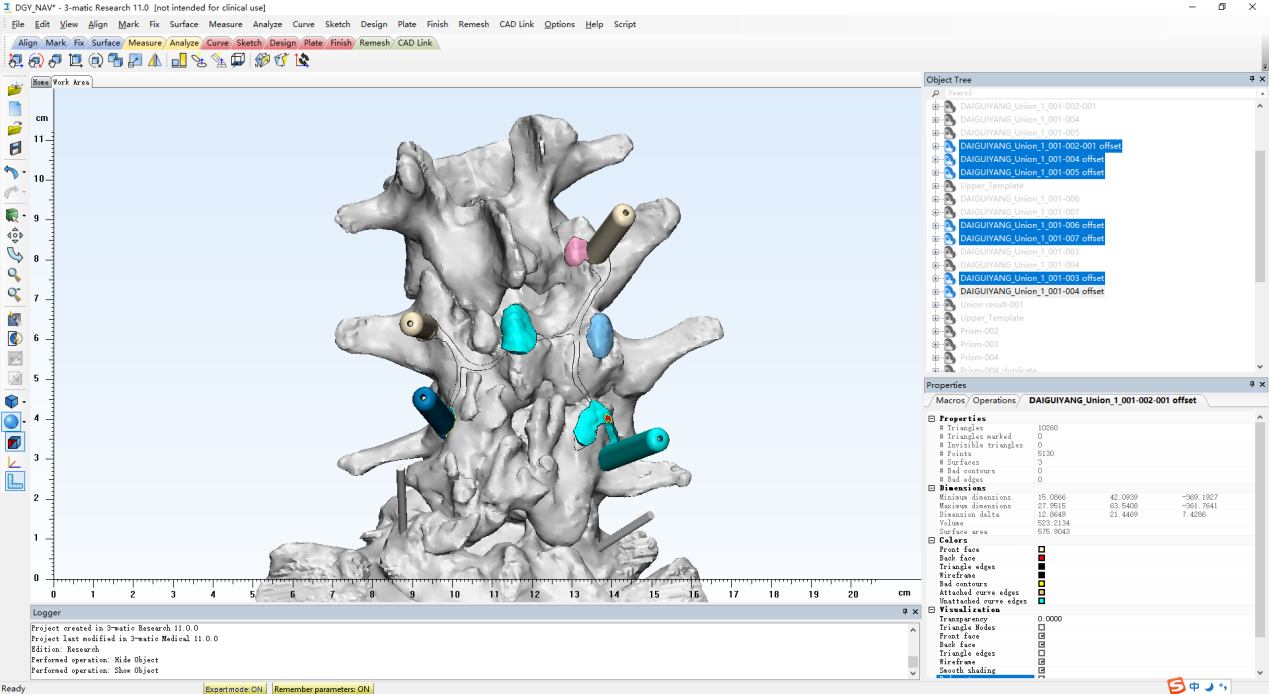


**B**


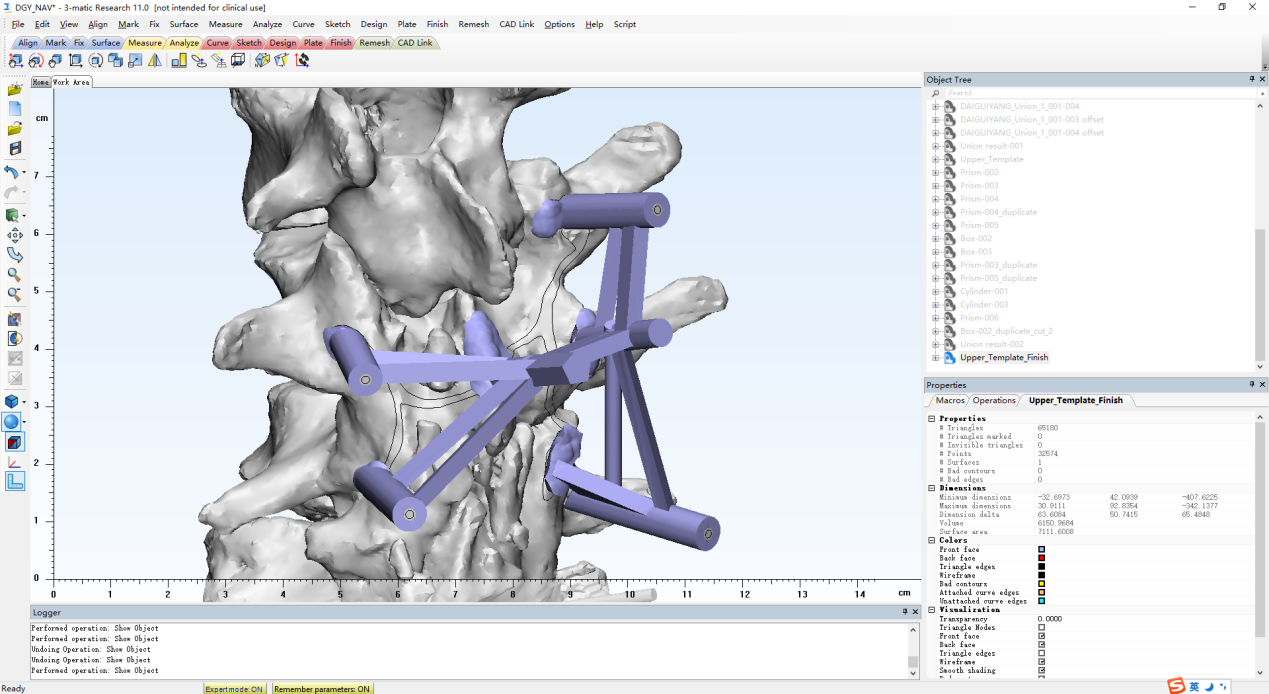


**Supplementary Figure 4.** Navigation template data in STL format were imported into Magics 20.03 3D printing pre-processing software for correction.


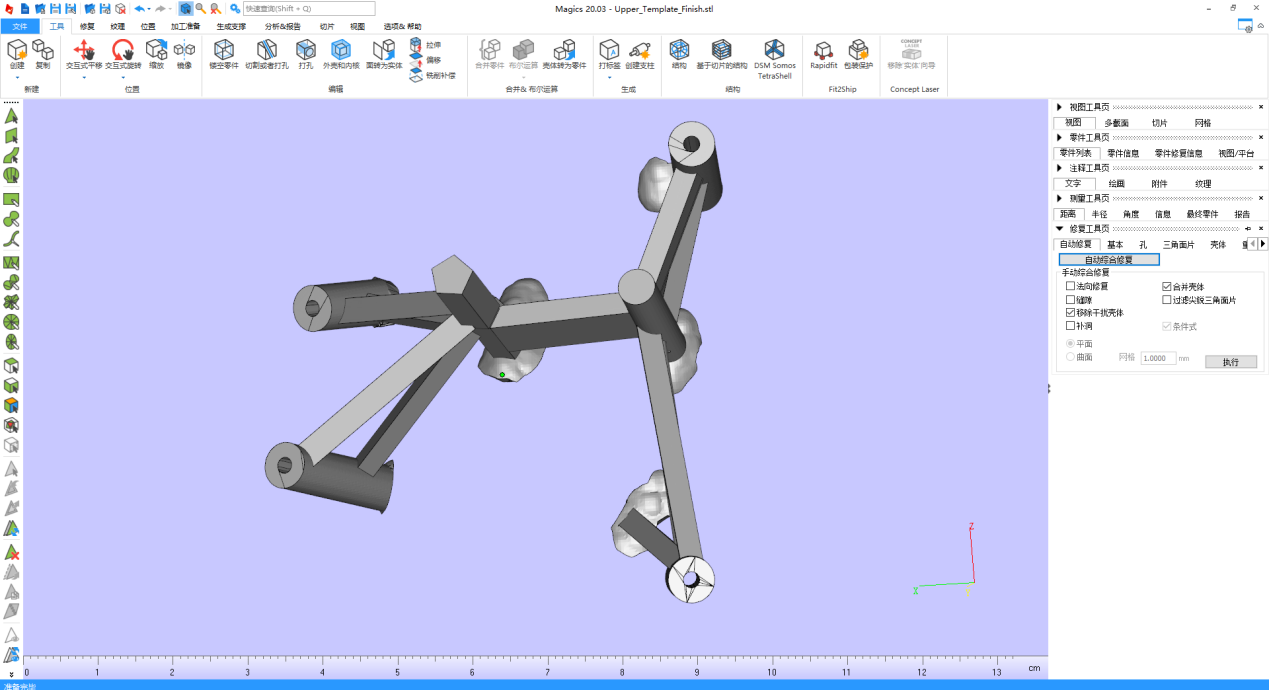

Supplement: Supplementary file 1 — Additional file 1: Supplementary Figure 1. Computed tomography (CT) data were converted to DICOM files by the PACS system, and then were imported into Mimics 19.0 software. The reconstructed model was obtained through 3D reconstruction calculation. Supplementary Figure 2. Four 2.2-mm diameter cylinders were generated using the Analyze module to simulate Kirschner wires for screw placement. The ideal path of the posterior pedicle screws was preliminarily designed (A), and the cylinders and the reconstructed model were fitted together (B). Supplementary Figure 3. A) Data were imported into 3-Matics software, the sites for positioning sites for guide plates were selected on the surface of the posterior spinous processes, lamina, and lateral mass of the vertebral bodies, and the guide plates were generated. B) After all connections were made, all local guide plates were integrated into a navigation template. Supplementary Figure 4. Navigation template data in STL format were imported into Magics 20.03 3D printing pre-processing software for correction.. [file 12891_2021_4892_MOESM1_ESM.docx]
